# Is choline kinase alpha a drug target for obesity?

**DOI:** 10.3389/fendo.2024.1492753

**Published:** 2024-11-06

**Authors:** Juan Carlos Lacal, Salam A. Ibrahim, Tahl Zimmerman

**Affiliations:** ^1^ Department of Metabolic & Immune Diseases, Instituto de Investigaciones Biomédicas, Agencia Estatal Consejo Superior de Investigaciones Científicas, Madrid, Spain; ^2^ Food and Nutritional Sciences Program, Department of Family and Consumer Sciences, North Carolina Agricultural and Technical University, Greensboro, NC, United States; ^3^ Biomedical Sciences Program, High Point University, One University Parkway, High Point, NC, United States

**Keywords:** choline, choline kinase, thermogenesis, adipogenesis, obesity

## Abstract

Choline kinase alpha (ChoKα) is a therapeutic target being developed for a variety of diseases, from cancer to rheumatoid arthritis and from parasites to bacterial infections. Nevertheless, the therapeutic potential of this drug target seems not exhausted and may end up as a possible solution for a larger variety of conditions. Here we present our working model for how ChoKα could play a role in obesity and for how drugs being developed as therapeutics for other diseases using ChoKα as a target, could be repurposed as prophylactic treatments for obesity. We also present preliminary observations in support of our model.

## Introduction

Choline kinase alpha (ChoKα) catalyzes the conversion of choline to phosphocholine (PCho) via the transfer of a phosphate group from ATP to choline. The formation of PCho is the first step in the Kennedy pathway, which is responsible for generating phosphatidylcholine, a critical cell membrane component. In its role in the production of the cell membrane, ChoKα functions as a mediator of cell growth and division. In keeping with this, it is upregulated in many types of cancer and has been proposed as a target for cancer therapy (1). Many inhibitors have been developed that can block the activity of human ChoKα (hChoKα) and other ChoKs, such as those found in parasites and bacteria. Therefore, ChoK inhibitors (ChoKIs) are promising therapeutics, not only for cancer (2), but also for malaria and other parasite-based diseases and even infectious disease (3–6). This family of inhibitors are also promising therapeutics for autoimmune diseases, such as rheumatoid arthritis, and several inflammatory conditions (1). One ChoKI, RSM-932A, has been developed to the point of reaching stage I clinical trials for patients with advanced tumors (7). Importantly, ChoKα inhibitors have been demonstrated to have very favorable toxicity profiles (8). These results suggests that research into ChoKI-based therapy is beginning to bear fruit (1). Nevertheless, the therapeutic potential of ChoKα as a drug target has not been fully explored. Further applications may be found in other conditions, such as obesity. Here we provide for the first time a rationale and preliminary evidence to support this new concept enlarging the applications of ChoKα inhibitors under development.

Obesity has become an epidemic in the US (9) and in developed countries overall (10). Obesity refers to the abnormal accumulation of adipose tissue and a subsequent increase in body weight. This condition is still not fully understood but it is, in part, a consequence of a chronic imbalance between the intake and expenditure of energy. This condition is also a consequence of the dysregulation of the appetite control system, sedentary behaviors, and even choice of food products (which are often a consequence of socioeconomic factors, as well as genetic causes) (11). Long-term obesity is associated with chronic diseases, including cardiovascular disease and diabetes (12). While lifestyle modifications are the ideal for managing body weight, alternative interventions such as nutritional supplements or pharmaceuticals should be explored to decrease the overall prevalence of obesity. While several effective options are available on the market today, some, such as Ozempic, have been associated with a significant range of side effects, including suicidal thoughts (13). Therefore, there is a need to develop safer alternatives.

Brown adipose tissue (BAT) is a type of adipose tissue that can be modulated to reduce weight by increasing energy expenditure (14). BAT expends energy by carrying out nonshivering thermogenesis (15). As a first step, AMPK activation leads to a chain of transcriptional events that leads to expression of Uncoupling protein 1 (UCP1) in BAT. UCP1 facilitates heat generation by leaking protons into the mitochondrial matrix, which leads to an increase in energy expenditure. This process uncouples oxidative phosphorylation from ATP synthesis (16). Therefore AMPK activators, such as resveratrol (17) and quercetin (18), have the potential to treat obesity by activating UCP1 and increasing energy expenditure.

ChoKα inhibition is known to activate AMPK (8). In addition, the AMPK activator quercetin is known to inhibit ChoKα (19), suggesting that ChoKα may lie upstream of the AMPK pathway that activates thermogenesis. Nevertheless, the potential of ChoKα inhibitors to treat obesity via activation of thermogenesis has remained unexplored.

Here we present our hypothesis that ChoKα is a putative target for the treatment of obesity. We also briefly discuss preliminary results and our working model, as well as future perspectives.

## Hypothesis

There are two major types of adipocytes: white adipocytes and brown adipocytes. White adipocytes store energy as fat when energy intake overtakes energy expenditure. Brown/beige adipocyte tissue (BAT) carry out non-shivering thermogenesis upon cold exposure and adrenergic stimulation; thus BAT promotes energy expenditure. The abnormal increase of white adipose tissue can occur by increasing the existing adipocyte cell size or the differentiation of new adipocytes. Adipogenesis is the proliferation and differentiation of adipocyte precursor cells into mature adipocytes, which accumulate in adipose tissues distributed throughout the body. Adipogenesis affects the overall number of adipocytes in the body; therefore modulating adipogenesis is one approach for treating obesity.

AMPK is a key energy sensor that regulates energy producing pathways in many tissues and is known to regulate the thermogenic genes in adipocytes (20). Activating the AMPK signaling pathway inhibits white adipogenesis, and promotes brown adipogenesis, and the “beiging” of white adipocytes. Both the inhibition of white adipocytes and the promotion of brown/beige adipocytes are therapeutic strategies for addressing obesity. BAT is highly active metabolically and contains mitochondria with elevated amounts of the thermogenic gene product UCP1. UCP1 mediates the uncoupling of the electron transport system in mitochondria, leading to a drop in the generation of ATP and consequently, increased thermogenesis and energy expenditure (21). Importantly, UCP1 is upregulated by AMPK via its downstream effectors SIRT1 and PGC-1α.

There is widespread research into the use of dietary compounds to increase energy expenditure via induction of brown adipose tissue thermogenesis (22). The AMPK pathway can be induced by dietary compounds such as capsaicin, resveratrol, and the flavonoid quercetin. There is extensive research into the use of AMP Kinase (AMPK)-activating compounds to induce weight loss by increasing energy expenditure via induction of brown adipose tissue (BAT) thermogenesis (20). As inducers of UCP1 mediated thermogenesis, these compounds have been shown to reduce obesity in high fat diet models of mice, both by activating BAT cells and by inducing the browning of white adipose tissue (23). The mechanism of action of many AMPK activator compounds is not completely understood, and the target proteins of these compounds are often unknown.

As an example, quercetin has been shown to activate many thermogenesis-related genes downstream of AMPK, including SIRT1, PPAR, γPGC-1α, UCP-1, PRDM16, CP51- α and PACC (24). Quercetin also reduces (via AMPK activation) IL1β in inflammation models (24). In cancer cell models, quercetin was shown to block the PI3K-Akt/PKB pathway (25). Importantly, AKT is a known negative regulator of AMPK (26).

ChoKα is a critical enzyme in the generation of phosphatidylcholine, the most abundant component of all cell membranes. In its role in the production of the cell membrane, ChoKα functions as a mediator of cell growth and division. ChoKα has been implicated in the AMPK pathway since pharmacological inhibition of ChoKα by small compounds or siRNAs leads to activation of AMPK (27), upregulation of PACC, downregulation of IL1β (28), and inactivation of the PI3K-Akt/PKB pathway (29). In addition, both siRNA knockdown and inhibition of ChoKα has been shown to induce uncoupling of the electron transport system in mitochondria (27). Choline depletion leads to a drop in ATP production in the mitochondria (30), and an upregulation of the AMPK effector PGC1α (31). As previously reported, inhibiting ChoKα leads a modification in the phospholipids composition of the mitochondria, causing the proton motive force to minimize and generate an imbalance in the AMP/ATP ratio, which in turn activates AMPK and the downstream pathway (27). Interestingly, choline deficiency attenuates weight gain in animal models (32). Taken together, this information supports the idea that ChoKα could play a role in BAT thermogenesis, and therefore, obesity via the AMPK signaling pathway.

Meanwhile, in a knockout model of PHOSPHO1, elevation in phosphocholine concentration has also been shown to activate thermogenesis (33). However, these authors describe in the same report an increased expression of PHOSPHO1 in BAT and WAT upon thermogenic activation. This latter observation would be more consistent with our results since it would reduce the levels of phosphocholine, in keeping with our proposal. Further research will need to be conducted to reconcile these apparently contradictory observations of a dual role of phosphocholine in the regulation of thermogenesis. In light of the model we propose, one possibility is that an accumulation of phosphocholine in cells would lead to product inhibition of ChoKa, resulting in an altered phospholipid composition in mitochondria membranes and the subsequent activation of AMPK. Product inhibition has been demonstrated for choline kinase (3). This could, in turn, function to activate AMPK similarly to the mechanism whereby a ChoKa inhibitor works.

ChoKα inhibition is also known to inactivate AKT, a negative regulator of AMPK. AMPK is known to activate SIRT1, which promotes the deacetylation of the transcription factors PRDM16 and PPARα/γ. These two transcription factors interact with a third component, PGC-1, and the PRDM16/PPARα/γ/PGC-1α complex together promote the transcription of genes leading to 3 characteristic qualities of BAT cells: 1) an increase in non-shivering thermogenesis, 2) an increase in mitochondrial biogenesis, and 3) an increase in energy expenditure observed in BAT cells (34). Similarly, AMPK can also directly enhance PGC1α activity by phosphorylation and an increase in UCP-1 thus increasing mitochondrial biogenesis and electron transport coupling, thermogenic genes, and increased energy expenditure.

The promise of research into AMPK activator compounds lies in the creation and commercialization of food products, supplements, and pharmaceuticals that would help address obesity. Nevertheless, the precise pathway whereby these compounds function, from protein target binding to gene expression is not understood. This lack of information limits the utility of these compounds, in terms of being prescribing them with precision and in a personalized fashion. We have postulated that these compounds function via inhibition of ChoKα.

Based on past reports (24–29) and preliminary data indicating the biochemical similarities between treatment with ChoKα inhibitors and AMPK activating dietary compounds, we hypothesized the following:

1. Some dietary compounds that activate the AMPK pathway may do so by binding and inhibiting ChoKα.
2. ChoKα inhibition leads to the activation of AMPK, BAT thermogenesis, and increased energy expenditure.
3. Inhibiting ChoKα should prevent accumulation of adipose tissue and weight gain.

**Figure 1:**
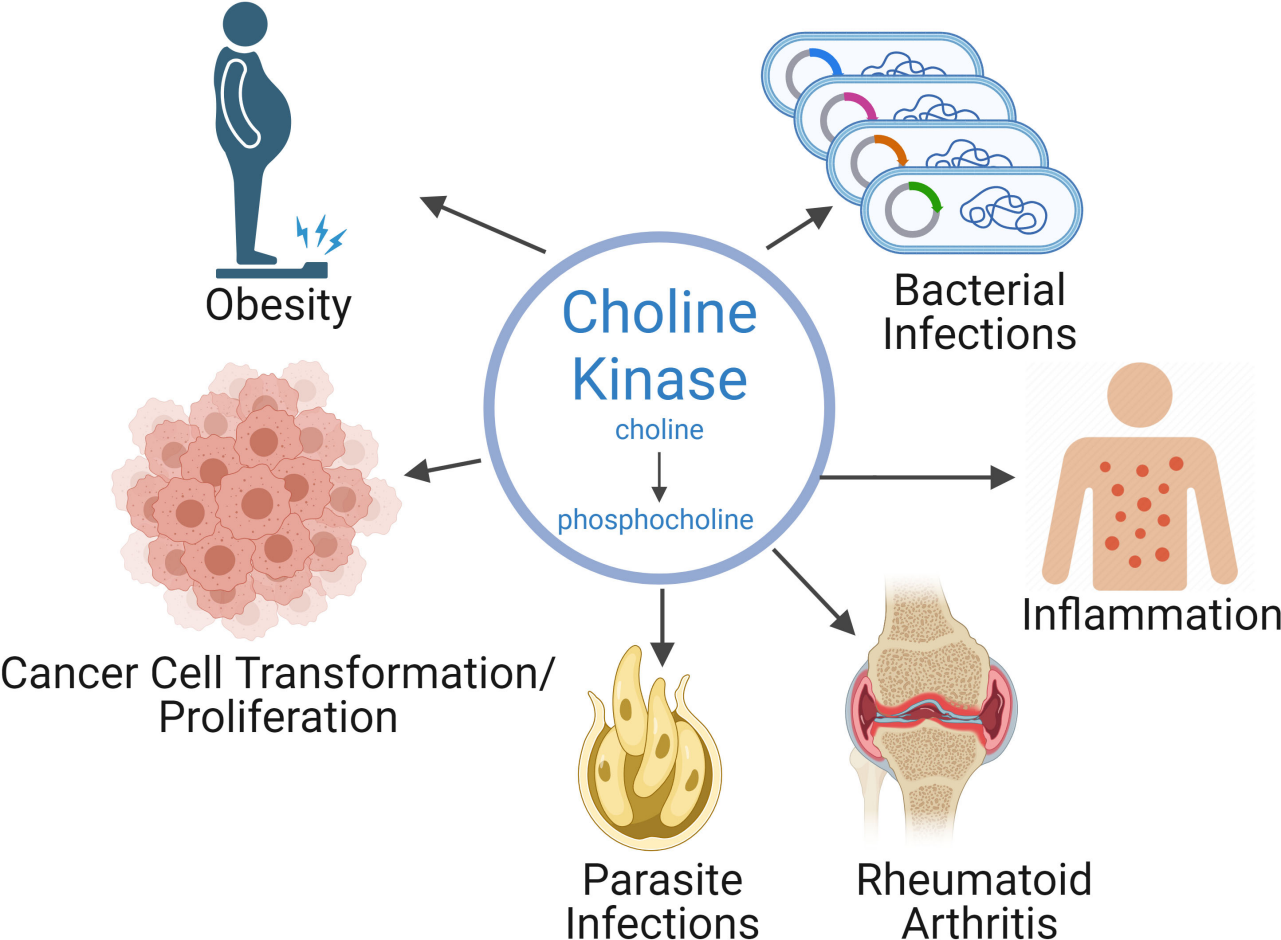
Choline kinase alpha plays a role in a variety of pathologies. As a consequence, inhibiting choline kinase is a therapeutic strategy for a wide range of pathologies, such as cancer, rheumatoid arthritis, inflammation, as well as parasitic and bacterial infections.

Inhibitors have been developed that can efficiently block the activity of hChoKα and other eukaryotic ChoKs, such as those found in parasites and bacteria. These inhibitors are promising therapeutics, not only for cancer, but also for malaria and other parasite-based diseases, some infectious diseases, and have shown as promising therapeutics for autoimmune diseases, rheumatoid arthritis, and inflammatory conditions (**Figure 1**) (1). One of our ChoKα inhibitors, RSM-932A, has been developed to the point of reaching stage I clinical trials for patients with advanced tumors (7) and have been demonstrated to have very favorable toxicity profiles (8).

## Preliminary data and model

In keeping with our proposal, we do find that treatment of 3T3-L1 cells with our ChoKα inhibitors does prevent differentiation into adipocytes and the expression of lipogenic genes. This information is consistent with the model that ChoKα is mediating the downstream processes via AMPK activation, since AMPK activation is known to prevent both differentiation and lipogenesis.

Additionally, our initial observations with mice fed a normal chow diet, followed by a concomitant treatment with both a high fat diet and ChoKα inhibitors suggests that these drugs are highly effective at preventing adipogenesis and weight gain. Similarly dramatic observations are seen with the murine treatment model where mice were fed a high fat diet followed by a concomitant regime of a high fat diet and ChoKα inhibitor treatment.

**Figure 2:**
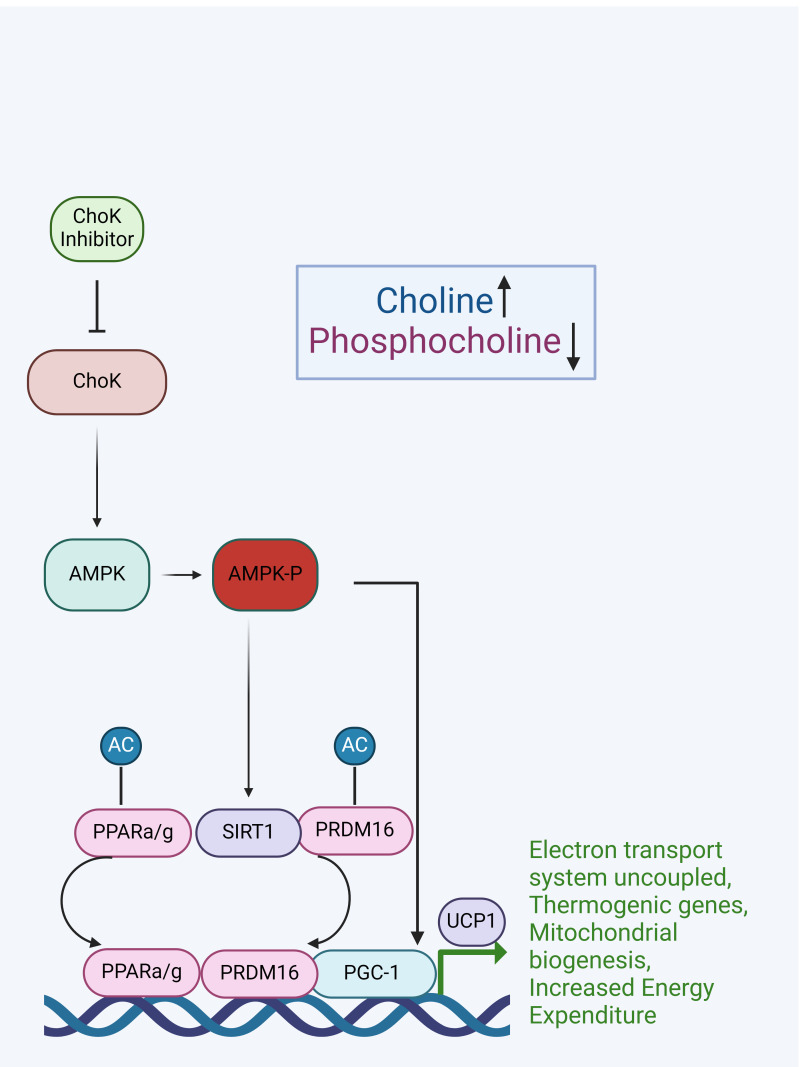
Model for how ChoKα inhibition would activate the AMPK–SIRT1–PGC-1α signaling pathway leading to WAT browning and the activation of thermogenic genes in BAT.

Thus, results with these well-established cellular and mouse models strongly support the hypothesis that ChoKα is indeed a prophylactic target for obesity. Therefore, known ChoKα inhibitors that demonstrated to be effective against other diseases could be repurposed for as a prophylactic for obesity. This is particularly supported in light of cell data indicating that inhibiting ChoKα blocks differentiation of adipocytic cells and attenuates lipogenesis, and that *in vivo* inhibition of ChoKα is effective to prevent obesity in mice, as we propose here. We suggest these effects could be mediated via AMPK activation and the subsequent activation of the thermogenic program in adipocytes. Our model for how ChoKα is implicated in thermogenesis is depicted in **Figure 2**.

## Conclusion

Developing ChoKα as a prophylactic target for obesity appears to be a promising avenue to follow. More work needs to be done to further elucidate the model.

## Data Availability

The datasets presented in this article are not readily available because this is unpublished data and therefore is not yet publicly available. Requests to access the datasets should be directed to tzimmerm@highpoint.edu.
