# Corrigendum: Is choline kinase alpha a drug target for obesity?

**DOI:** 10.3389/fendo.2025.1580595

**Published:** 2025-03-13

**Authors:** Juan Carlos Lacal, Salam A. Ibrahim, Tahl Zimmerman

**Affiliations:** ^1^ Department of Metabolic & Immune Diseases, Instituto de Investigaciones Biomédicas, Agencia Estatal Consejo Superior de Investigaciones Científicas, Madrid, Spain; ^2^ Food and Nutritional Sciences Program, Department of Family and Consumer Sciences, North Carolina Agricultural and Technical University, Greensboro, NC, United States; ^3^ Biomedical Sciences Program, High Point University, One University Parkway, High Point, NC, United States

**Keywords:** choline, choline kinase, thermogenesis, adipogenesis, obesity

In the published article, there was a mistake in the **Funding** statement. The authors mistakenly stated that no financial support was received for this research.

The correct statement is “The author(s) declare that financial support was received for the research, authorship, and/or publication of this article. This research was supported by Grant PID2020-116165RB-C21 funded by MCIN/AEI/10.13039/501100011033 to JL.”

The authors apologize for this error and state that this does not change the scientific conclusions of the article in any way. The original article has been updated.

